# Trustworthy Health-Related Tweets on Social Media in Saudi Arabia: Tweet Metadata Analysis

**DOI:** 10.2196/14731

**Published:** 2019-10-08

**Authors:** Yahya Albalawi, Nikola S Nikolov, Jim Buckley

**Affiliations:** 1 Department of Computer Science and Information Systems University of Limerick Limerick Ireland; 2 Department of Computer and Information Sciences College of Arts and Science University of Taibah Al-Ula Saudi Arabia; 3 The Irish Software Research Centre Lero University of Limerick Limerick Ireland

**Keywords:** social media, new media, misinformation, trustworthiness, dissemination, health communication

## Abstract

**Background:**

Social media platforms play a vital role in the dissemination of health information. However, evidence suggests that a high proportion of Twitter posts (ie, tweets) are not necessarily accurate, and many studies suggest that tweets do not need to be accurate, or at least evidence based, to receive traction. This is a dangerous combination in the sphere of health information.

**Objective:**

The first objective of this study is to examine health-related tweets originating from Saudi Arabia in terms of their accuracy. The second objective is to find factors that relate to the accuracy and dissemination of these tweets, thereby enabling the identification of ways to enhance the dissemination of accurate tweets. The initial findings from this study and methodological improvements will then be employed in a larger-scale study that will address these issues in more detail.

**Methods:**

A health lexicon was used to extract health-related tweets using the Twitter application programming interface and the results were further filtered manually. A total of 300 tweets were each labeled by two medical doctors; the doctors agreed that 109 tweets were either accurate or inaccurate. Other measures were taken from these tweets’ metadata to see if there was any relationship between the measures and either the accuracy or the dissemination of the tweets. The entire range of this metadata was analyzed using Python, version 3.6.5 (Python Software Foundation), to answer the research questions posed.

**Results:**

A total of 34 out of 109 tweets (31.2%) in the dataset used in this study were classified as untrustworthy health information. These came mainly from users with a non-health care background and social media accounts that had no corresponding physical (ie, organization) manifestation. Unsurprisingly, we found that traditionally trusted health sources were more likely to tweet accurate health information than other users. Likewise, these provisional results suggest that tweets posted in the morning are more trustworthy than tweets posted at night, possibly corresponding to official and casual posts, respectively. Our results also suggest that the crowd was quite good at identifying trustworthy information sources, as evidenced by the number of times a tweet’s author was tagged as *favorited* by the community.

**Conclusions:**

The results indicate some initially surprising factors that might correlate with the accuracy of tweets and their dissemination. For example, the time a tweet was posted correlated with its accuracy, which may reflect a difference between professional (ie, morning) and hobbyist (ie, evening) tweets. More surprisingly, tweets containing a kashida—a decorative element in Arabic writing used to justify the text within lines—were more likely to be disseminated through retweets. These findings will be further assessed using data analysis techniques on a much larger dataset in future work.

## Introduction

### Background

In recent years, there has been significant growth in the uptake of personal communication technologies around the world. This has been largely afforded by the widespread availability of social media (SM) and has been facilitated by the increase in mobile phone ownership. SM has become a valuable tool for communication and it has been utilized in many areas, such as education [1], marketing [2], and health communication [3]. For example, in the field of health communication, the US Centers for Disease Control and Prevention (CDC) [4] and local health departments in the United States [5] have used Twitter to communicate to people during epidemics. Another example is from the United Kingdom and Norway, where health authorities used Twitter to inform their citizens during the West African Ebola outbreak in 2014 and 2015 [6].

The use of SM can improve the nature of health communication as it speeds up the interaction between health care organizations, professionals, and patients [3]. Thus, various SM platforms and apps can play a vital role in health communication and in the promotion of good health [7]. Despite the advantages that SM potentially offers for health communication, it also faces certain challenges. For example, during a health crisis, there is only a limited amount of time for authorities to respond in an efficient way and inform people, while simultaneously helping to eliminate uncertainty on a topic. If this does not occur promptly, it is much more likely that rumors will spread, possibly through SM; when this happens, the negative effects of SM, such as confusion and misinformation, are the probable results [8].

Illustrative examples include the negative consequences experienced by Saudi Arabia and African countries during the Ebola and Middle East respiratory syndrome (MERS) outbreaks. In Saudi Arabia, SM rumors prevented some people from going to emergency departments when they were in an acute condition, resulting in the cancellation of their surgical procedures [9]. In Africa, rumors were found on SM that drinking a huge amount of salt water was a cure for Ebola; it has been reported that this may have caused the deaths of several people [10]. Notably, these misinformation issues seem to affect developing countries more deeply; studies have suggested that 4.5% of Twitter posts (ie, tweets) in the United States are misinformed [11], however, Oyeyemi et al [10] found evidence showing that 50% of tweets in West Africa are misinformed.

Three different studies conducted on health and different types of users reported that Twitter was the preferred platform among health professionals [12], medical students [13], and diabetic patients [14]. In addition, most health-related studies on SM focus on the English-speaking population and the United States [15,16] and not on other cultures. Specifically, Hagg et al [17] reported the absence of literature analyzing SM data for health-related purposes in the Middle East. This is particularly surprising because recent statistics from Statista indicate that Saudi Arabia has the fourth-highest number of Twitter users in the world [18]. Furthermore, when assessing the ratio between Twitter users and populations for each country [19], Saudi Arabia has the highest number of users on Twitter relative to its population. These findings are also supported by other researchers who reported on the elevated prevalence of Twitter usage in Saudi Arabia [20,21]. That being said, only one study—Alnemer et al—has analyzed Saudi health tweets [22].

Given the likely cultural differences between Twitter use in the West and in the Middle East, it seemed important to assess health-related tweets in a Middle Eastern country. Given the prevalence of Twitter use in Saudi Arabia, that seemed like an appropriate country to choose. Hence, this study focuses exclusively on Saudi Arabia.

While a number of tweet characteristics have been assessed for accuracy of the information on SM, the foremost characteristic of interest in this regard has been the source of the tweet. Intuitively, one would anticipate that tweets from health professionals would be more trustworthy; however, this is an open question. A study by Alnemer et al [22] found that 50% of Saudi health professionals’ tweets were not evidence based. In addition, this study only includes tweets that were posted by accounts with more than 45,000 followers. The relatively high number of followers suggests that these account holders might be considered opinion leaders in their domain of expertise, which, in this case, is health.

These findings question the accuracy of health professionals’ tweets, which is a worrying result considering that people are traditionally more likely to trust users who are physicians, health organizations, and pharmacists [23-27]. While these sources are trusted, evidence shows that they are not necessarily trustworthy (ie, accurate); Alnemer et al [22] suggest that, even if sources are traditionally trusted, there is a high possibility that they include inaccurate (ie, untrustworthy) information.

A few methods and tools for detecting misinformation on SM, particularly during a health crisis, have been proposed and are usually focused on specific topics and diseases (eg, Ebola and Zika) [28,29]. They typically strive to identify the characteristics of misinformation, while neglecting the factors that indicate trustworthy tweets.

In this study, tweets are considered to be trustworthy if they are accurate and are considered to be untrustworthy if they are inaccurate. This position is similar to Yin et al [30], who state that a website is trustworthy if it provides correct information, and information is likely to be true if it is provided by a trustworthy website. Likewise, Zhao et al [31], who developed a topic model to estimate the trustworthiness of the news on Twitter, defined a trustworthy tweet as one that refers to things that really happened. Similarly, this paper considers a tweet as trustworthy if it contains accurate health information and if the process of evaluating the accuracy of tweets is introduced in the methods section.

The perspective we take in our work is to focus on determining the factors that correlate with the trustworthiness of health information tweets as well as the factors that affect the dissemination of those tweets. This work has been undertaken in order to determine how SM might be effectively oriented toward the dissemination of trustworthy health information.

We assess the trustworthiness of tweets originating from traditionally trusted health sources and examine the relationships between attributes (>100) of a tweet and its trustworthiness.

Tweets do not need to be accurate or evidence based to receive traction. In their study, Nastasi et al [32] noted that scientifically inaccurate health tweets were retweeted in the same manner as accurate tweets. That work also indicated the need to study the dissemination metrics of tweets in order to find factors that correlate with high dissemination. Consequently, as a second objective, we will assess the factors that correlate with larger dissemination of health tweets.

### Prior Work

SM data from Facebook, Instagram, YouTube, and Twitter have been used to understand people’s attitudes and behaviors in sharing and consuming information related to specific health issues, such as vaccinations, abortions, posttraumatic stress, and cancer [33-38]. Facebook has been used by both private health stakeholders and government agencies to engage with the public [39,40]; studies have provided understanding by analyzing Facebook’s timelines [40] and health agencies’ accounts on Facebook [39].

Although Facebook is the most popular overall platform, the most popular SM platform to study health is Twitter [41-43], as evidenced by studies included in different systematic reviews of topics related to health and social media [44-47]. This focus may be due to the complexity of Facebook data and its unavailability due to privacy restrictions [41].

Most health studies on Twitter collect their data by using specific keywords [28,29,48-52] or from tweets authored by specific health stakeholders, such as health organizations [5,22,53-58]. However, it appears that most studies that analyze Twitter for health using specific keywords have not analyzed the types of users who post the tweets [28,29,48-52]; it is known from other studies that there are different types of users and they share different types of information or hold different attitudes toward specific health issues [59-62]. For example, a number of studies performed tweet extraction using keywords to identify public concerns during the Zika outbreak [48-52]. Besides being limited to a particular outbreak, these studies did not analyze the interplay between public concerns and the types of users and did not address the factors that make a tweet trustworthy.

A notable study of health-related tweets in Arabic was the one performed by Alnemer et al [22], which manually analyzed the accuracy of tweets authored by preselected health accounts. Their results suggested that governmental institutions are more likely to tweet accurate information than are health professionals or other institutions (ie, physicians, dieticians, and nongovernmental and unofficial health institutions). They reported that 80% of the observed governmental institutes’ tweets consisted of accurate health information, followed by physicians (60%). However, the overall accuracy of the tweets, over all observed accounts, was 50%. These findings suggest that even if SM users have health expertise, it cannot be taken for granted that their tweets provide accurate health information. This line of investigation can easily be extended to nonhealth users. Alnemer et al [22] did not examine the characteristics of a tweet that may correlate with its trustworthiness.

In terms of trustworthiness, a number of classifiers for health-related tweets in English have been proposed. For example, Ghenai and Mejova [58] proposed a classifier to detect health rumors on Twitter limited to the Zika virus. A limitation of this study is the fact that their classifier was trained on a limited number of rumors, identified as such by information on external non-SM websites. In addition, annotators who labeled the tweets as misinformed were not health experts. In another study, Ghenai and Mejova [29] focused on the detection of users tweeting or propagating misinformation about cancer, excluding social bots and organizational accounts.

However, social bots have also been considered as a possible source for health misinformation on SM [63,64]. For example, Allem et al [64] analyzed tweets in regard to e-cigarette discussions and found that social bots may support misinformation on SM in regard to e-cigarette cessation. In their study, they emphasized the importance of distinguishing between social bots and real users. Similar to Allem et al [64], Broniatowski [63] analyzed tweets specifically to understand how bots promote online health content in regard to vaccine-related messages and found that social bots were one of the possible sources for antivaccine advice on SM.

As suggested above, in terms of social bots and misinformation on SM, previous research has emphasized the importance of distinguishing social bots from real users, particularly when the intent is to assess views held by users who are not bots [64,65]. However, this is not an easy task, as some social bots might mimic user behavior [66]. Social bots might introduce themselves as individual accounts with locations and photos for their profiles [64,66]. Furthermore, some organizations use social bots to disseminate information, which makes it hard to distinguish between it being the *opinions of the bot* or that of their organization and classifying according to their organization might be difficult [67,68]. In the work presented here, users were classified as they introduced themselves and it was that classification that was analyzed in terms of the accuracy of information they portrayed.

Kalyanam et al [28] analyzed the association between hashtags and the credibility of tweets related to the Ebola outbreak. They defined credible tweets as those with hashtags that indicated origin from well-known governmental agencies or other authoritative sources (eg, #cdc or #cnn) and speculative tweets as those with hashtags that indicated the spread of fear, rumor, scam, or humor. It was determined that almost 25% of the analyzed tweets were speculative. Their findings suggested that verified users were more likely to interact with credible hashtags; on average, the number of followers for accounts that posted tweets with credible hashtags was 7000, compared to 2700 for accounts that posted tweets with speculative hashtags in their dataset. Kalyanam et al relied on hashtags, without evaluation of the information carried by the tweets. That is, it is unclear whether tweets classified as credible really contain accurate or trustworthy information.

In terms of identifying influential users on SM, Albalawi and Sixsmith [69] applied six different tools to identify the most influential Twitter users in Saudi Arabia. First, they used the apps Tweepar and SocialBaker, which reveal the most influential users per country on Twitter. With the influential users being identified, they collated four Twitter influence scores via the following: Social Authority by Moz [70], PeerIndex [71], Kred [72], and Klout [73]. However, within the scope of their study, they did not consider health in isolation and they did not analyze the accuracy of the tweets.

Wong et al [5] analyzed tweets sent by 287 local health departments (LHDs) during the Ebola epidemic in the United States. They found that 70% of the LHDs tweeted at least once about Ebola and that Twitter had become a frequent tool used by LHDs during this particular epidemic. Regarding the dissemination of tweets, one of their findings was that the presence of hashtags and links was highly correlated with the messages being retweeted. Similarly, Suh et al [74] also reported that tweets containing hashtags and links were more likely to be retweeted. They did not consider the impact of the type of users on retweeting. Furthermore, the analyzed tweets were on randomly selected topics, unrelated to the health care domain.

The research results summarized in this section suggest that there is a lack of comprehensive studies on the accuracy of health-related tweets on SM. Specifically, to the best of our knowledge, there are no results that determine the factors that make a health-related tweet trustworthy in general, besides it being authored by a credible institution. In addition, there are no studies on the general factors that affect the dissemination of trustworthy health-related tweets, besides the credibility of the author and the presence of hashtags and links. We believe that the identification of such factors may help health organizations in better disseminating trustworthy information during an outbreak-related health crisis. In particular, there is a lack of studies on health-related tweets in Arabic; this makes such a study a priority, considering the high popularity of Twitter in the Arab world.

Hence, the work presented in this paper addresses the following questions:

1. How can the trustworthiness of health care stakeholders’ tweets be identified from the tweets’ features?
  - What proportion of trustworthy health-related tweets come from the following sources: health professionals, health organizations, and authorities?
  - What are the other characteristics associated with trustworthy health-related tweets?
2. What are the factors that contribute to the wider dissemination of health care-related tweets that could possibly be used to make accurate health information dominant over other related information on SM?
  - Does the trustworthy nature of health-related tweets increase their dissemination?
  - What other factors contribute to the dissemination of health care-related tweets?

The structure of this paper is as follows. First, the paper introduces the empirical design for identifying the factors affecting the trustworthiness and dissemination of health-related tweets. Second, it presents the results of our work and highlights, in particular, the findings that correspond to the research questions listed above. Finally, this paper ends by discussing the future directions of our research and the possible implications of the results.

## Methods

### Overview

This work utilized a standard text analytics methodology, which incorporated the following steps:

1. We developed a health lexicon using two different methods.
2. By means of this health lexicon, we extracted health tweets using the Twitter application programming interface (API).
3. From the remaining tweets, we manually refined tweets related to health using two annotators.
4. Medical professionals manually labeled the remaining tweets as either accurate or inaccurate.
5. We extracted features from the labeled dataset. These included attributes of the tweets as well as attributes of the user profiles for the users who authored the tweets. In this paper, only aggregated user data is presented for ethical reasons.
6. We analyzed the labeled dataset to provide preliminary answers to the research questions outlined in the previous section.

**Figure 1:**
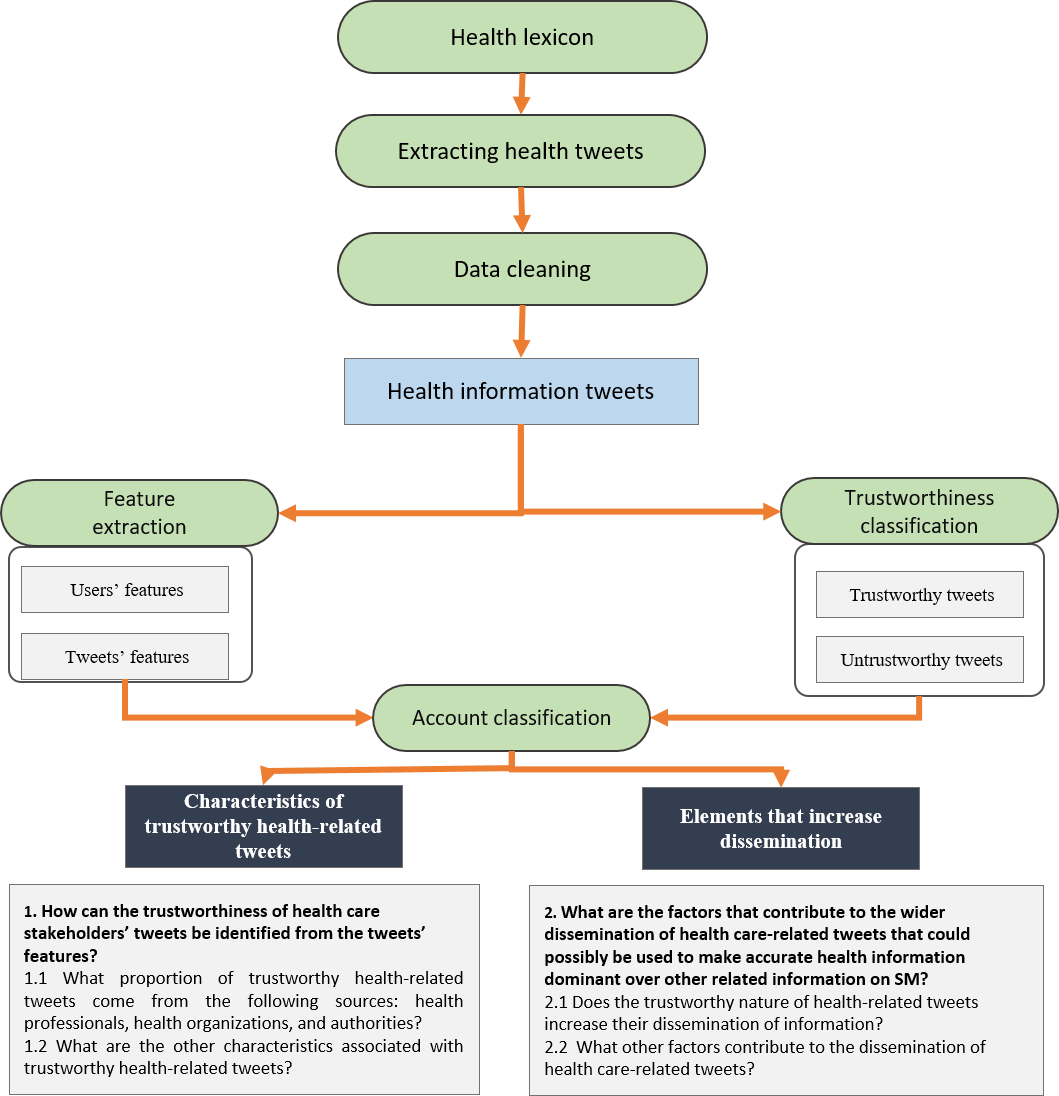
Overview of the research methodology. SM: social media.

The outlined methodology is presented in Figure 1. The sections that follow explain each step in detail.

### Construction of the Health Lexicon

In order to identify health-related tweets, a health lexicon was created. It is important to note that the incorrect selection of indicative health keywords could bias the results [75]. Therefore, two separate methods of analysis were utilized to generate this lexicon. The first method consisted of asking three medical doctors with active Twitter accounts to provide 100 health-related words. The doctors we asked are skilled in different disciplines and differ from each other in their age, background, and gender. They were asked to provide an initial list of health-related words that they think would be used in tweets related to health. The second method involved the usage of 110 health care keywords that were independently identified by an annotator with a college degree in linguistics. As this study concentrates on Saudi Arabia, the annotator identified these keywords by examining a set of tweets with geolocation that indicated a Saudi Arabian origin, although people who enable geolocation are likely to represent a specific demographic group [76,77]. As such, the words chosen by the annotator might have limited generalizability with respect to the wider demographic group. Thus, the annotator also reviewed health-related accounts and hashtags to identify different health-related words. A complete list of keywords is attached in Multimedia Appendix 1.

These two methods were combined in an attempt to construct a health lexicon that is as unbiased as possible.

### Data Cleaning

Using the lexicon developed as described in the previous section, it was then possible to extract Twitter data for the main part of our work. The Twitter API does not allow users to extract tweets more than a week old [78]. To reduce the impact of this limitation, it was decided that we would extract two datasets. The first dataset was extracted on May 18, 2018, and the second dataset on August 7, 2018. Table 1 describes the characteristics of each dataset.

**Table 1:** Characteristics of the datasets.

Characteristics	First dataset, n (%)	Second dataset, n (%)
Total tweets	209,345 (100)	196,670 (100)
Original tweets	57,794 (27.61)	39,454 (20.06)
Reply tweets	28,329 (13.53)	32,470 (16.51)
Retweeted tweets	123,222 (58.86)	124,746 (63.43)

Reply tweets were difficult to evaluate by annotators, due to a lack of contextual information. As a result, these tweets were removed. It was also necessary to remove all retweeted tweets due to redundancy.

The tweet extraction process resulted in an accumulation of 97,248 tweets: 57,794 from the first dataset and 39,454 from the second. Using the random method in Python, version 3.6.5 (Python Software Foundation), a sample of 2800 tweets was selected from the set for use in a prototype study. Even though the lexicon suggested that all of the 97,248 tweets were health related, a manual examination of the tweets showed that this was not necessarily so. Thus, two annotators were employed to filter out tweets unrelated to health from the sample of 2800 tweets. The guidelines for the annotators were as follows:

1. Tweets that describe any function of the body, such as enzymes, organs, or diseases, should be retained.
2. Tweets that give advice or information about supplements, drugs, physical activity, or food and link it to people’s health, such as how vitamins, food, and drugs affect people’s health, should be retained.

Based on an internal discussion among the three authors of this study, these guidelines were derived from Bobicey and Sokolova’s ontology for personal health information [79]. The terms were derived from concepts in their ontology and were considered by all three authors as the most indicative of health-related material.

Each annotator labeled 60% of the tweets, with 10% of the tweets (n=280) labeled by both annotators in order to check the reliability of the analysis. The Cohen kappa statistic for interrater reliability [80] was then calculated, resulting in a value of .872, which indicates excellent agreement between the annotators.

Out of the 2800 tweets, only 552 tweets (19.71%) were labeled as health related. Out of these 552 tweets, 180 tweets (32.6%) originating from the first dataset were selected, in addition to 120 tweets (21.7%) originating from the second dataset. By doing so, it was possible for us to retain the proportion of tweets in the originally collected datasets.

### Trustworthiness Classification

Once the previous processes had been completed, 10 medical doctors were asked to manually classify the tweets into the following categories:

1. Accurate health information.
2. Inaccurate health information.
3. Not sure about the accuracy.

The *not sure* option was given to the doctors to avoid forcing them to make a decision on tweets if they did not have enough relevant health knowledge to accurately evaluate them or if the tweets were ambiguous.

**Figure 2:**
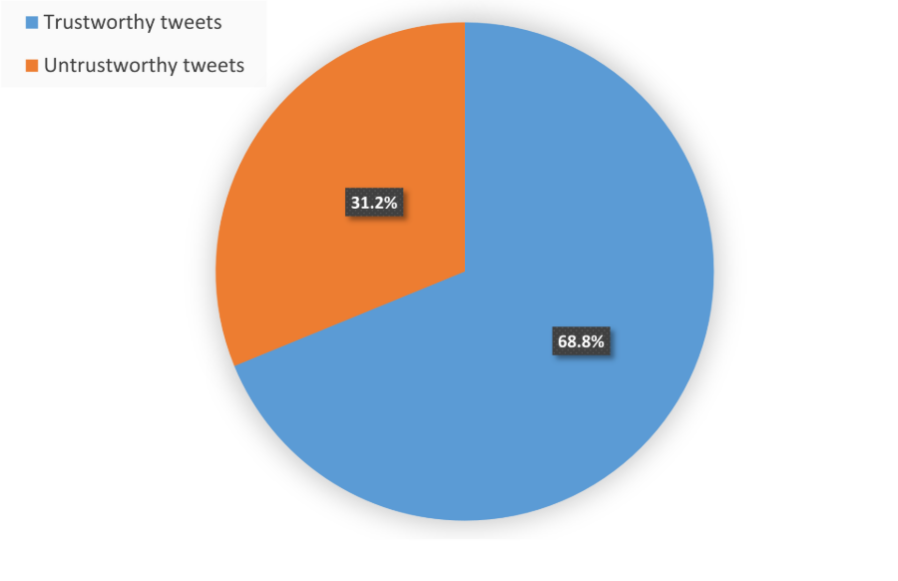
Proportion of trustworthy (n=75) and untrustworthy (n=34) tweets in the sample of 109 labeled, health-related tweets.

A total of 10 Google forms were created, each containing 30 tweets. A link to each form was sent to two doctors by email. In order to achieve high reliability with respect to the accuracy and inaccuracy, we excluded any tweets that a doctor labeled as *not sure*. In addition, we excluded any tweets where the two doctors coding them disagreed on their accuracy. This resulted in 109 labeled tweets in the spreadsheet, 75 (68.8%) of which were labeled as trustworthy (ie, tweets that both doctors labeled as accurate) and 34 of (31.2%) of which were labeled as untrustworthy (ie, tweets that both doctors labeled as inaccurate). The information was then transferred to a spreadsheet and analyzed using Python for descriptive statistics. For the statistical tests, we used the R package, version 3.4.0 (The R Foundation) [81]. The output of this process is illustrated in Figure 2.

### Feature Extraction

#### Overview

To answer the research questions, it was necessary to determine the features of the tweets as well as their level of trustworthiness. The features of the tweets were categorized into two types: tweet features and user features.

These features, whether on the user level or the tweet level, may provide data that is useful in classifying the types of users or identifying the credibility of the tweets for different topics [82-86].

#### Tweet Features

Tweet features were extracted directly from the tweets. These included whether or not the tweet was retweeted (ie, as a dissemination measure) as well as various linguistic characteristics of the tweets, such as the number of words and the number of characters in each tweet. Tweet features also included other properties of the tweets, such as URLs contained in the tweet, the time of the tweets, and hashtags. Most of these features have been used by other researchers; however, the linguistic features identified in most other studies were analyzed for Latin-derived words. Thus, more features were added after reviewing literature related to Arabic natural language processing. These features are as follows:

1. Tashkeel: the presence of a tashkeel in the tweet. The tashkeel is a special Arabic character written in the text to represent missing vowels [87].
2. Kashida: the presence of a kashida in the tweet. The kashida is a decorative element in Arabic writing used for justifying the text [88].

In addition, similar to Castillo et al [82], we examined different types of punctuation marks in tweets and their relationship to information credibility. This was based on our insight that people who used punctuation in their tweets appeared more thorough and that this might be associated with greater accuracy and dissemination.

A complete list of tweet features is provided in Multimedia Appendix 2.

#### User Features

#### Overview

Tweets come with metadata that provide basic profile information about the user who posted the tweet, such as screen name, number of friends, number of followers, favorite count, retweet count, and age of the account. In addition, there are cumulative tweeting characteristics for each user. To derive this data, we used the Twitter API to extract another 200 tweets per author by the authors of the 109 labeled tweets. The tweet number of 200 has been suggested as sufficient for extracting user features [83,89]. The user features were categorized into four groups, which will be described in the following sections. A complete list of these features is provided in Multimedia Appendix 2.

#### Activity and Connectedness Features

These features include metrics that measure how active the user is, such as how often the user replies to other users or how often the user retweets [86].

#### User Linguistic Features

These features include measures of how often the user uses unique hashtags, the average number of hashtags used in tweets, and the mean number of words in the user’s tweets. Such linguistic features have been used in other studies to assess information credibility on Twitter [82] and to classify users on SM [83,90].

#### User Time Features

These features deal with the temporal aspect of the user's tweets. For example, previous research examined the day of the week when tweets were posted to determine if the day is linked to the credibility of news [82]. In the study presented here, more features were added, such as the preferred time of the day—morning, evening, and night—for users to tweet and whether the tweet was posted during weekdays or weekends [91].

#### User Popularity Features

These features indicate the popularity of the users, such as the number of followers per user as well as how often users’ tweets were retweeted [86].

### Account Classification

Accounts were classified according to the following criteria [53,59,61,62,69,92]:

1. Does the account holder have a health background?
2. Is the account that of an individual or nonindividual?
3. If the account holder is a nonindividual, does the account represent a physical authority or is it an exclusively SM-based account?

Hence, by the end of this process, it was expected that the following categories of user accounts could be analyzed:

1. Individual health accounts.
2. Individual nonhealth accounts.
3. Health organization accounts.
4. Nonhealth organization accounts.
5. Exclusively SM-based accounts.
6. Users whose profiles cannot be extracted and, therefore, remain unknown.

Two annotators classified the types of tweets in the dataset based on author accounts and disagreed on seven users. They met to explain their opinions to each other. Finally, after a discussion they agreed on the categories of five of these seven users. An expert in health communication on SM was consulted—a surgeon with a PhD in Health Promotion in New Media—to classify the final two users. In addition, there were about 10 accounts for which profile data could not be extracted; these accounts were classified as *unknown*.

### Data Analysis

Lancaster et al [93] recommended that the execution of a pilot study should primarily rely on descriptive and distribution statistics as results. For the continuous variables, it was decided to present the median number, as the median is not affected by the outliers. For the categorical variables, it was decided to employ a statistical test to determine preliminary results. As our data is nonparametric, the Mann-Whitney-Wilcoxon (MWW) test was utilized to establish statistical significance. The MWW test is considered appropriate for nonparametric data and for when the two samples are from different populations (ie, in our case, accurate tweets and inaccurate tweets) and of different sizes [94].

For categorical variables, the Fisher exact test was also used, which is suitable for a small sample of less than 1000 [94]. In the study presented here, there were two categorical variables with more than two values: the type of author and the times at which tweets were posted. Typically, the Fisher exact test does not test for statistical significance for a contingency table larger than 2×2; however, in R it is possible to calculate the *P* value for larger contingency tables, hence R was used here. The mechanism that allows for the calculation of the *P* value is based on the work of Mehat and Petal [95] and Clarkson et al [96].

## Results

In this section, the most promising results are presented, which indicate factors that may demonstrate the trustworthiness and untrustworthiness of health-related tweets. Overall, more than 100 tweet-level and user-level features in a dataset of 109 tweets were explored; these tweets were labeled as either accurate and trustworthy or inaccurate and untrustworthy.

An initial analysis of the tweet-level features indicates that trustworthy health tweets were significantly more likely to have an author that is a member of a list (ie, a curated group of Twitter accounts) (*P*=.05). Although not significant, trustworthy tweets seemed more likely to be favorited by others (*P*=.06).

In contrast, Table 2 suggests that untrustworthy health tweets were more likely to have URLs embedded in them (*P*=.03). Specifically, 24% (8/34) of the untrustworthy tweets had URLs, compared to 8% (6/75) of trustworthy tweets. A total of 4 out of 8 (50%) of the URLs cited in inaccurate tweets referred to news websites, while 2 out of 8 (25%) referred to blogs.

**Table 2:** Most promising tweet features to help distinguish the accuracy level of tweets.

Metric	Description	Trustworthy tweets (N=75), n (%)	Untrustworthy tweets (N=34), n (%)	*P* value^a^
URLs	The tweet contains a URL	6 (8)	8 (24)	.03
Listed	The author is listed	53 (71)	17 (50)	.051
Favorited	The tweet is favorited	46 (61)	14 (41)	.06
Hashtags	The tweet contains a hashtag	20 (27)	4 (12)	.13
Tashkeel	The tweet contains a tashkeel	22 (29)	5 (15)	.15
Exclamation mark	The tweet contains “!”	3 (4)	4 (12)	.20
Semicolon	The tweet contains “;”	42 (56)	14 (41)	.21
Retweeted	The tweet was retweeted	43 (57)	15 (44)	.22
Kashida	The tweet contains a kashida	6 (31)	8 (24)	.49

^a^*P* values were calculated using the Fisher exact test with *P*≤.05 indicating statistical significance.

The user-level features were also analyzed (see Table 3). The analysis revealed the worrying trend that users who had a lower *number of followees* (F3) were more likely to tweet accurate health tweets (*P*<.001). More encouragingly, the popularity measure of *number of times that author’s tweets are favorited* (FT2) was associated with trustworthiness, suggesting that accurate tweets were recognized as such. Interestingly, authors who tended to retweet tweets that had hashtags (RMH5) also tended to tweet trustworthy tweets, although the *P* value is not quite significant in that case (*P*=.06).

**Table 3:** Metrics for users who tweet accurate information versus users who tweet inaccurate information.

Metric	Description	Trustworthy tweets, median (SD)	Untrustworthy tweets, median (SD)	*P* value^a^
F3	Followees count	75.5 (2517)	891 (12,952)	<.001
FT2	Number of times author’s tweets are favorited (ie, favorite-author tags)	281.5 (76,054)	46 (2454)	.01
RMH5	Unique hashtag count in tweets that were retweeted by the author	6 (24.2)	1 (26.6)	.06
OT3	Number of hashtags in the author‘s tweets	18 (144.4)	6 (64.5)	.09
RM1	Number of retweeted tweets by the author where the user mentioned other users	20 (76.3)	13 (49.7)	.09
FT6	Number of original tweets posted by the author that are favorited	55 (60.5)	26 (58.7)	.11
SSI	Ratio of original tweets posted by the author to tweets retweeted by the author	4.51 (61.6)	8.94 (93.8)	.11
MH5	Unique keyword count in hashtags set in original tweets posted by the author	7 (46.1)	3 (22.9)	.14
RP1	Number of reply-to tweets posted by the author	21 (47)	3 (54.1)	.20
RMM5	Unique mentions in retweeted tweets by the author	7 (26.1)	4 (24)	.43
M1	Number of tweets where the author mentioned other users	18.5 (61.6)	10 (66.6)	.51
FT1	Number of tweets favorited by the author	179 (1202.6)	83 (14,073)	.84

^a^*P* values were calculated using the Mann-Whitney-Wilcoxon (MWW) test with *P*≤.05 indicating statistical significance.

**Figure 3:**
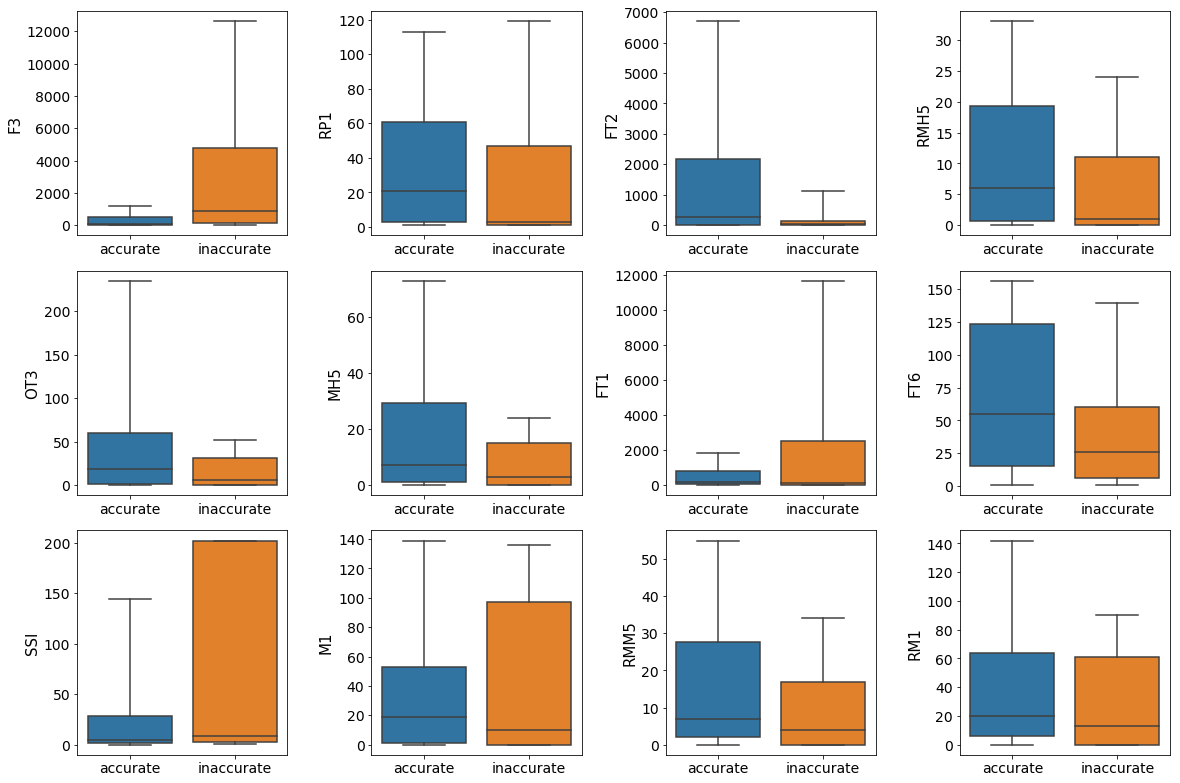
Boxplots for the features most closely correlating with the trustworthiness of tweets; outliers outside the 90th percentile were excluded. F3: number of followees; FT1: number of tweets favorited by the author; FT2: number of times that author’s tweets are favorited; FT6: number of original tweets posted by the author that are favorited; M1: number of tweets where the author mentioned other users; MH5: unique keyword count in hashtags set in original tweets posted by the author; OT3: number of hashtags in the author‘s tweets; RM1: number of retweeted tweets by the author where the user mentioned other users; RMH5: hashtag count in tweets that were retweeted by the author; RMM5: unique mentions in retweeted tweets by the author; RP1: number of reply-to tweets posted by the author; SSI: ratio of original tweets posted by the author to tweets retweeted by the author.

Figure 3 shows the boxplots for the user features, with outliers beyond 90% of the data excluded. This figure suggests that there may be other metrics associated with trustworthiness given a larger dataset, such as the *number of tweets favorited by the author* (FT1), the *number of original tweets posted by the author that are favorited* (FT6), and the *ratio of original tweets posted by the author to tweets retweeted by the author* (SSI).

**Figure 4:**
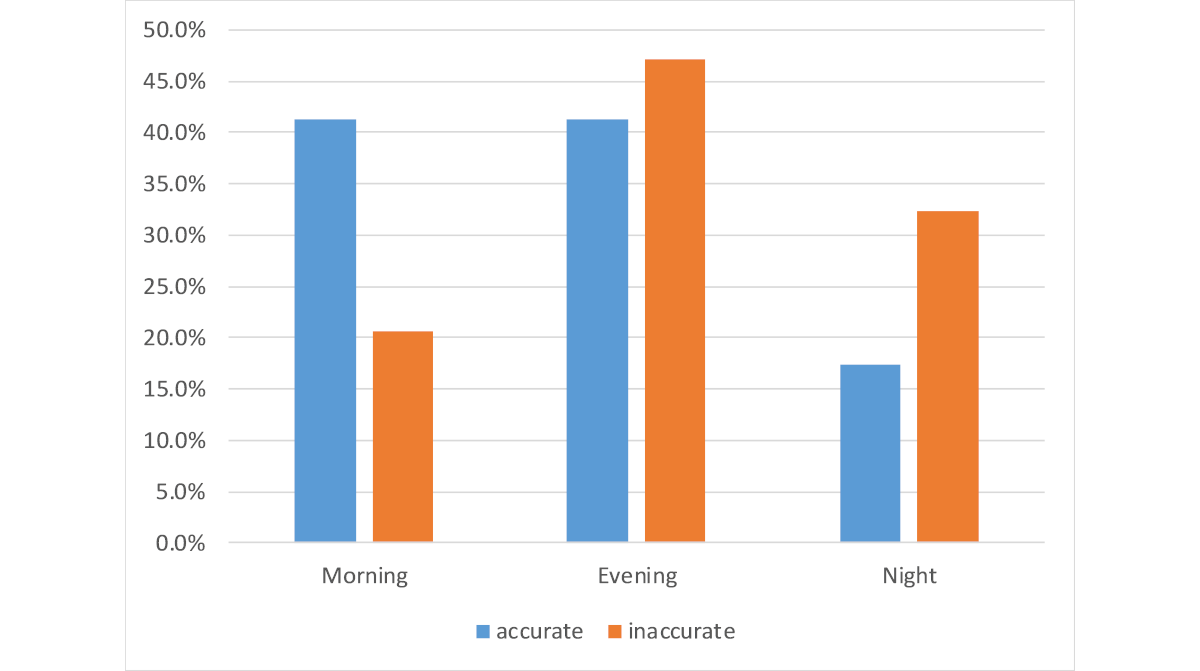
Distribution of accurate and inaccurate tweets per time of day in three categories: morning (6 am-2 pm), evening (2 pm-10 pm), and night (10 pm-6 am).

As per Figure 4, regarding the timing metrics, it appears that the majority of the trustworthy tweets were posted in the morning, while most untrustworthy tweets were posted either in the evening or at night. The associated Fisher test is borderline, with a *P* value of .06. However, when comparing only two groups—the morning tweets and the night tweets—statistical significance is achieved with a *P* value of .04. This result aligns with our insight that professional tweets are posted during the daytime and more informal tweets are posted at nighttime.

**Figure 5:**
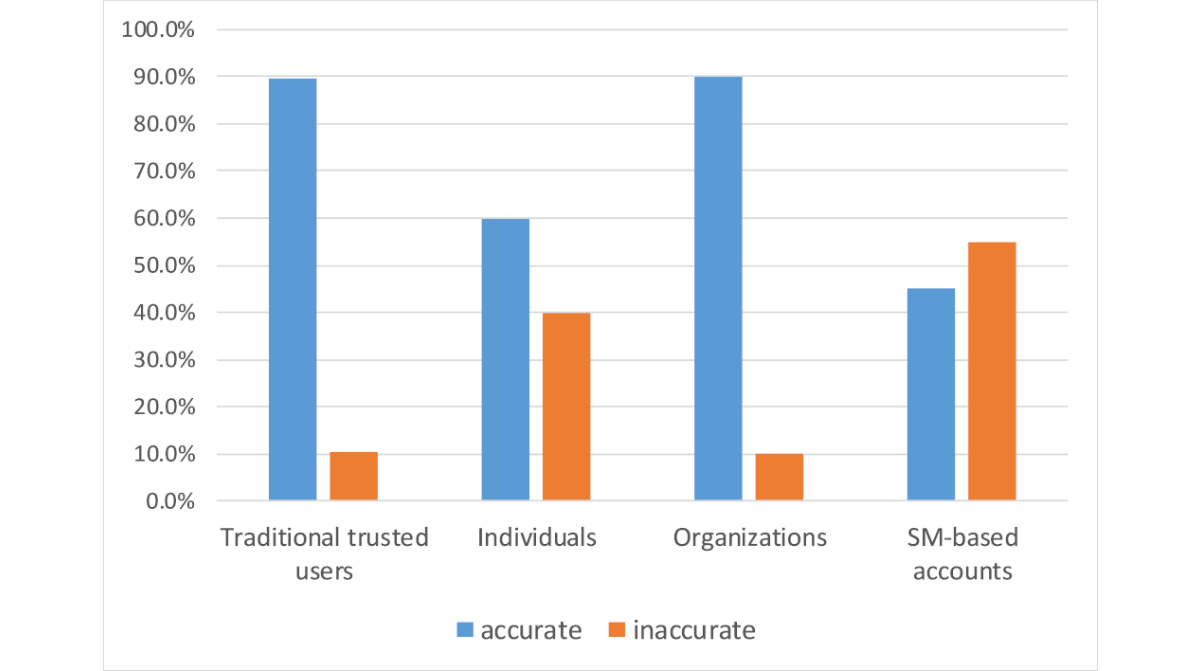
Accuracy of the tweets posted by each author type. SM: social media.

As can be seen in Figure 5, sources classified as organization accounts were more likely to tweet accurate information, followed by sources traditionally considered as trusted users (ie, health professionals, health authorities, and health organization accounts). However, there were only 10 tweets from organization accounts, one of which was from an organization unrelated to health care. Overall, traditionally trusted users are considered the most trustworthy source in the dataset, as there were 34 tweets from them and only 4 (12%) were considered inaccurate. The least trustworthy category in the dataset includes the users with exclusively SM-based accounts.

**Figure 6:**
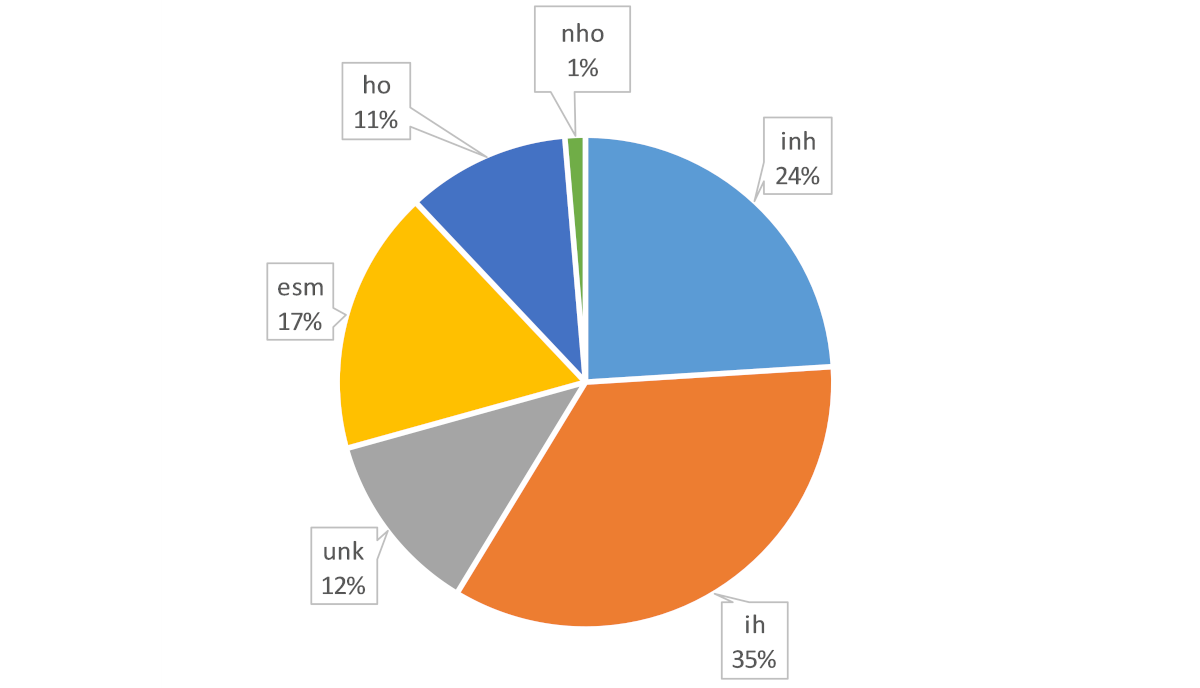
Distribution of the authors of accurate (ie, trustworthy) health-related tweets. esm: exclusively social media-based accounts; ho: health organization accounts; ih: individual health accounts; inh: individual nonhealth accounts; nho: nonhealth organization accounts; unk: users whose profile cannot be extracted and, therefore, remains unknown.

**Figure 7:**
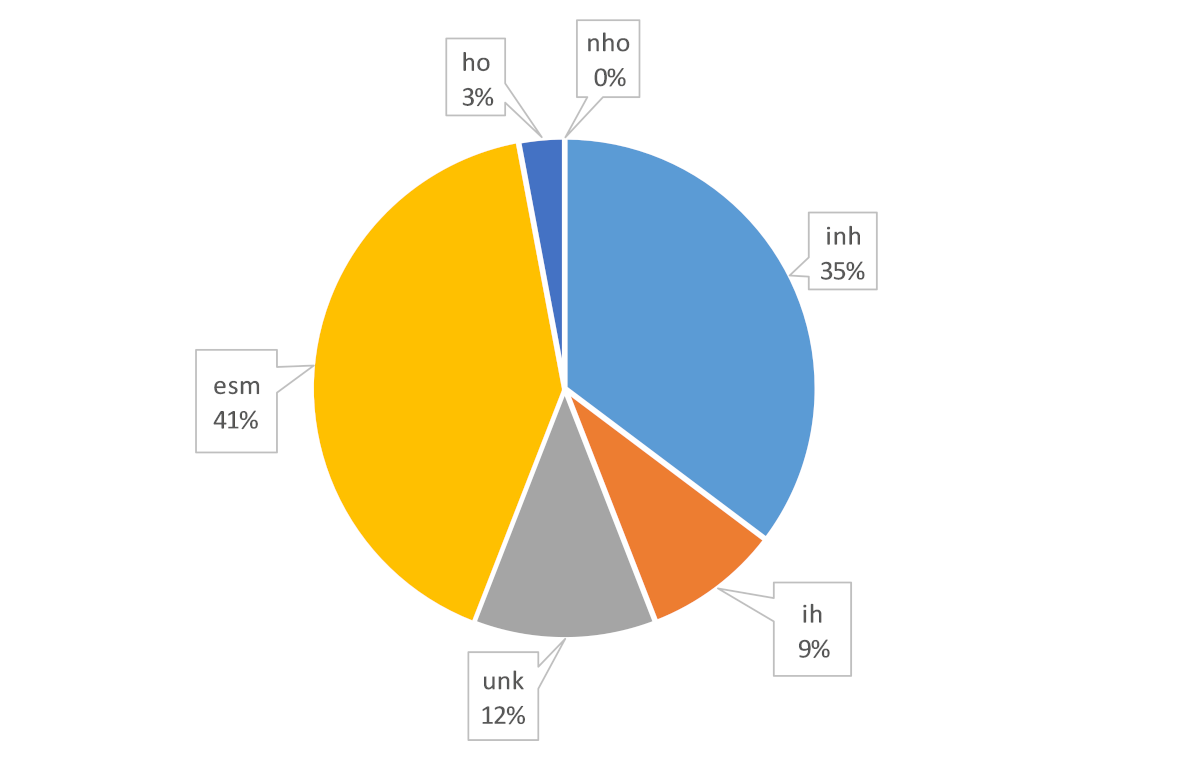
Distribution of the authors of inaccurate (ie, untrustworthy) health-related tweets. esm: exclusively social media-based accounts; ho: health organization accounts; ih: individual health accounts; inh: individual nonhealth accounts; nho: nonhealth organization accounts; unk: users whose profile cannot be extracted and, therefore, remains unknown.

When the backgrounds of individual users were considered, those with a background in health care (ie, health professionals) seemed to tweet accurate health information and were less likely to tweet inaccurate health information (see Figures 6 and 7); they posted 29 out of 109 tweets (26.6%). Individuals from a non-health care background were core players in terms of the volume of tweets, with 18 out of 30 (60%) of their tweets labeled as trustworthy. Overall, however, they authored 12 out of 34 (35%) of the untrustworthy tweets and only 18 out of 75 (24%) of the trustworthy tweets.

Another interesting finding is that exclusively SM-based accounts seemed to tweet much more inaccurate health information than other account types. Therefore, to summarize, trusted health accounts (ie, health care organizations and professional health care individuals) were more likely to tweet trustworthy health information than were other types of accounts. Individual users were the main players in terms of the volume of health information on SM, but the high volume did not correlate with trustworthiness. The Fisher exact test indicated statistical significance between the type of the user who posted a tweet and the accuracy of the tweet (*P*=.04).

**Figure 8:**
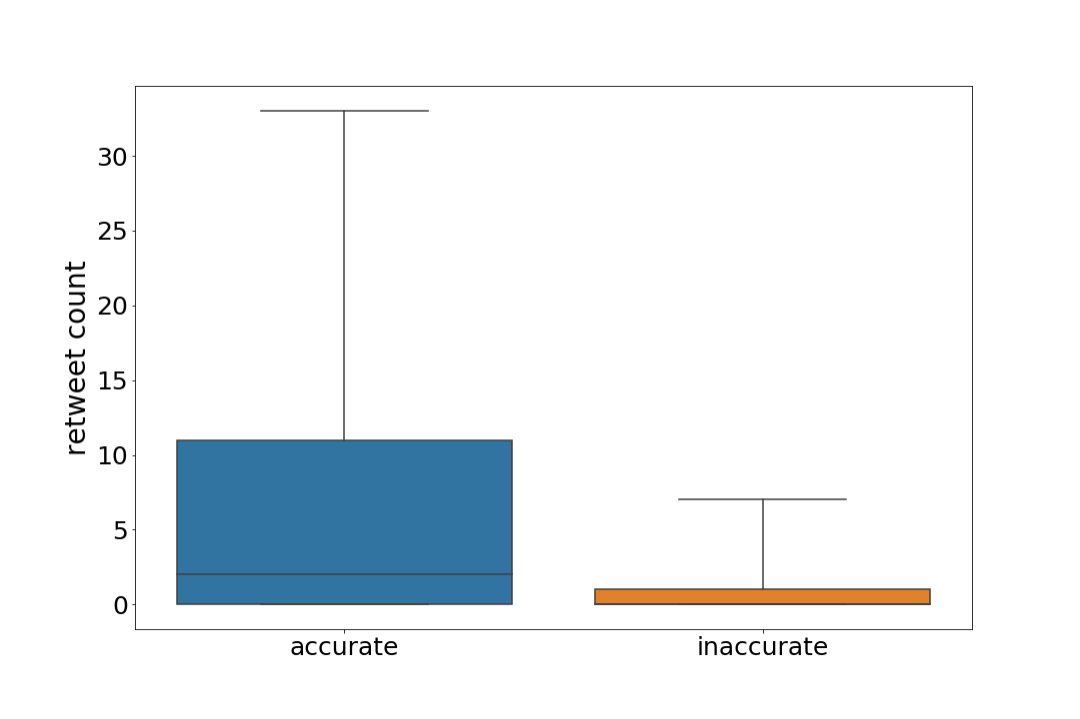
Retweeted counts of accurate and trustworthy versus inaccurate tweets in our dataset; outliers outside the 90th percentile were excluded (*P*=.043).

To address the question of whether the trustworthiness of tweets increases their dissemination, a retweeting metric was examined. According to Suh et al [74], retweeting is the key mechanism for information dissemination on Twitter. The results of our study indicate that trustworthy health information was slightly more likely to be retweeted than inaccurate health information, but the difference was not significant. Specifically, 43 out of 75 (57%) of the accurate tweets were retweeted compared to 15 out of 34 (44%) of the inaccurate tweets being retweeted. This is in line with the findings in other studies, which found that health information does not need to be accurate in order to be disseminated [32]. However, when accurate health-related tweets were retweeted, they were more likely to be further retweeted than inaccurate health-related tweets, as shown in Figure 8.

The provisional findings suggest that tweets with embedded commas, listed authors, or marked as favorite were also associated with dissemination (*P*<.001), as indicated in Table 4, which also lists a few other factors we examined. The only factor that was clearly counter-indicative of dissemination was the presence of a URL in the tweet, with only 5.2% of these tweets being retweeted.

Table 5 shows statistical significance for most of the popularity metrics.

**Table 4:** Features for retweeted tweets versus unretweeted tweets.

Metric	Description	Retweeted tweets (N=58), n (%)	Unretweeted tweets (N=51), n (%)	*P* value^a^
Listed	The author is listed	50 (86)	20 (39)	<.001
Favorited	The tweet is favorited	51 (88)	9 (18)	<.001
Comma	The tweet contains a comma	25 (43)	9 (18)	.01
Kashida	The tweet contains a kashida	22 (38)	9 (18)	.02
URLs	The tweet contains a URL	3 (5)	11 (22)	.02
Tashkeel	The tweet contains a tashkeel	19 (33)	8 (16)	.04
Tweet accuracy	The tweet is accurate	43 (74)	32 (63)	.22
Hashtags	The tweet contains a hashtag	14 (24)	10 (20)	.64

^a^*P* values were calculated using the Fisher exact test with *P*<.05 indicating statistical significance.

**Table 5:** Metrics for users who tweeted accurate information versus users whose tweets were retweeted.

Metric	Description	Retweeted tweets, median (SD)	Unretweeted tweets, median (SD)	*P* value^a^
FT2	Number of times that author’s tweets are favorited	857.5 (8366)	25 (121)	<.001
TFF	Ratio of followers to followees (F1/F3)	81.6 (18,950)	1.09 (22,728)	<.001
Listed count	Number of lists where the user is member	29.5 (912.1)	1 (99)	<.001
RT2	Number of author’s tweets retweeted by other users	79.5 (59.8)	9 (25.5)	<.001
FT6	Number of original tweets posted by the author that are favorited	77 (61.5)	17 (42.3)	<.001
M2	Number of unique users mentioned by the author	16 (43.1)	3 (23.5)	.052
RP1	Number of reply-to tweets posted by the author	24.5 (53)	4 (42.2)	.08
SSI	Ratio of original tweets posted by the author to tweets retweeted by the author	3.83 (63.4)	21.11 (87.6)	.11
M1	Number of tweets where the author mentioned other users	18.5 (67.4)	3 (47.6)	.12
RM1	Number of retweeted tweets by the author where the user mentioned other users	20 (78.3)	7 (56.2)	.12
RT1	Number of tweets that the author retweeted	19.5 (49.1)	7 (54.4)	.14
OT3	Number of hashtags in the author’s tweets	20 (142.5)	9 (103)	.26

^a^The *P* value was calculated using the Mann-Whitney-Wilcoxon (MWW) test with *P*<.05 indicating statistical significance.

**Figure 9:**
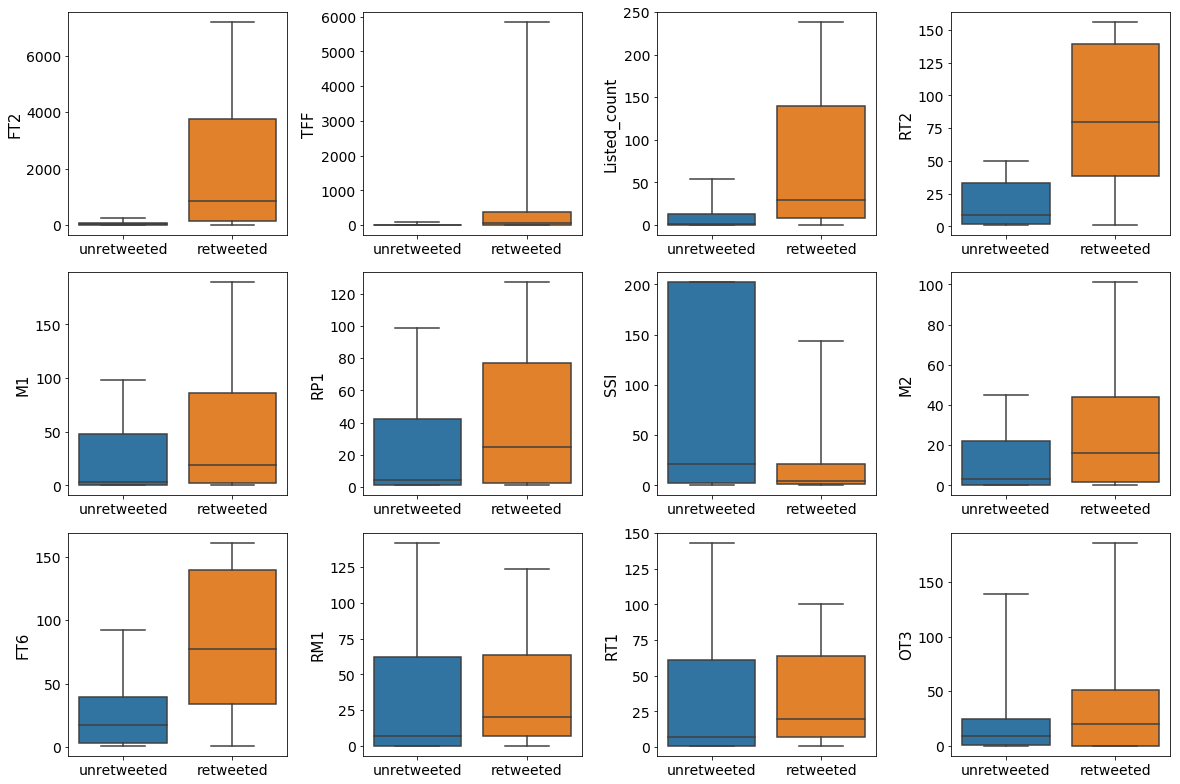
Boxplots for the features most closely correlated to dissemination; outliers outside the 90th percentile were excluded. FT2: number of times that author’s tweets are favorited; FT6: number of original tweets posted by the author that are favorited; M1: number of tweets where the author mentioned other users; M2: number of unique users mentioned by the author; OT3: number of hashtags in the author‘s tweets; RM1: number of retweeted tweets by the author where the user mentioned other users; RP1: number of reply-to tweets posted by the author; RT1: number of tweets that the author retweeted; RT2: number of author’s tweets retweeted by other users; SSI: ratio of original tweets posted by the author to tweets retweeted by the author; TFF: ratio of followers to followees.

The user features *ratio of followers to followees* (TFF), FT2, *number of author’s tweets retweeted by other users* (RT2), FT6, and listed count appear to have strong associations with dissemination, as shown in Figure 9. This comes as no surprise, as these features are typically considered to be popularity metrics. Furthermore, the results, as indicated in Table 5, show that users who mention other users/posters of tweets (M2) are more likely to have their tweets retweeted.

## Discussion

### Principal Findings

In terms of individual health tweets, the results of this study do not agree with those of Alnemer et al [22], in that they suggested that 50% of the tweets were not evidence based. However, Alnemer et al labeled every tweet in their dataset as either accurate or inaccurate, while in this study, tweets for which not all annotators agreed were excluded. This difference in annotation may explain the differences in the results. Nevertheless, this contradiction indicates the importance of conducting future research to explain these distinctions.

In this preliminary analysis, a group of users linked to the accuracy of the health information was identified, indicating that trusted health users are more likely to tweet trustworthy health information than inaccurate health information. This association is supported by the findings of Medlock et al [26].

Nevertheless, a high proportion of tweets from individuals with no health background were also found to be accurate. This observation suggests the existence of a subgroup of trustworthy SM accounts. The isolation of such a subgroup might be possible through the identification of other characteristics.

Both Wong et al [5] and Suh et al [74] reported that interacting with hashtags was linked to dissemination, while this study provided no clear evidence of such a relationship. Instead, we found that the more a user interacted with other users (ie, *number of times that author’s tweets are favorited* [FT2]), the more likely it was that their tweets were accurate. This finding suggests that trustworthy users have more influence than other users, as FT2 is considered to be an influence metric [86].

Interestingly, the data revealed that most of the accurate health tweets were posted in the morning, while most of the inaccurate tweets were posted at night. This disparity may occur because health professionals may tweet accurate health information while they are at work, possibly as part of their job, while less-trustworthy tweets are more likely posted at night when nonprofessionals are more likely to give an opinion.

In addition, there is no clear answer as to whether trustworthiness is linked to dissemination, because trustworthy tweets were only slightly more likely to be retweeted. However, when considering the retweet count, accurate tweets were more likely to be retweeted more frequently, as shown in Figure 8. These preliminary results suggest that there is an association between trustworthiness and the ultimate dissemination of the tweets.

Similar findings were also noted by Kalyanam et al [28]; it raises the question as to why trustworthy tweets are more likely to be retweeted more frequently once they are retweeted. One interpretation might be that there are thresholds for followers that can be exceeded and once they are exceeded, the author might have a certain leverage for their tweets to be retweeted more [97]. However, neither this study nor that of Kalyanam et al looked at self-retweeting specifically. This practice is known to be common in microblogs, such as in circumstances where users retweet to win prizes. Surprisingly, tweets with embedded commas and kashidas were retweeted more, suggesting that correct punctuation may be perceived as a sign of accuracy.

Moreover, some tweet metrics appeared to be linked to both dissemination and trustworthiness; for instance, tweets that embedded the tashkeel were more likely to be retweeted and indicated a trend of possibly being more trustworthy, while tweets that embedded URLs were less likely to be retweeted and trustworthy. These findings contradict those of Wong et al [5], who analyzed specific health accounts and found that URLs in tweets were associated with dissemination.

In regard to the source of the URLs cited in inaccurate tweets, our findings indicated that news websites were the most cited (50%). This is in line with Ghenai and Mejova [58], who found news websites were the most cited sources in inaccurate tweets (39% of the URLs).

Our findings, as shown in Tables 2 and 4, suggested that the language characteristics of the tweets might be associated with both dissemination and trustworthiness. At a high level, this may suggest that the style in which tweets are written is also linked to dissemination and trustworthiness. At a low level, some of these features are language specific; for example, the tashkeel is used in Arabic but does not exist in Latin languages. This specificity indicates the need to take language type into account when designing any future study. The tashkeel was not tested for significance in trustworthy tweets; as *P* was equal to .10, these results should be considered for future studies.

In future work, we will seek to develop a machine learning model for classifying health-related tweets as either trustworthy or not trustworthy. To do this, we aim to employ a larger dataset and to evaluate the usefulness of a larger set of features as predictors. Some of these features may include measures for the linguistic ability of a user. The extraction of additional features may also require the development of additional machine learning models, such as models for topic detection in order to measure, for example, how often a user tweets about health.

### Internal Validity

Due to the limited data size, this study provided preliminary findings on the relationship between the variables studied. In addition, this study did not establish any causal relationship between variables, only correlations.

### External Validity

The selection of data in this study was limited to health-related tweets in the Arabic language on Twitter. In addition, we collected tweets from a limited time period: two periods of 7 days. Ultimately, we analyzed a small number of tweets and, as a result, we cannot presume generalization for the findings of this study.

We did not include any reply tweets in the analysis; therefore, our results cannot be generalized to interaction-type communication on SM (eg, if the user posts a direct health question to another user). Our results only refer to initial tweets.

Although we used two methods in developing a lexicon, we cannot claim that the lexicon is totally representative of the population. Secondly, in this study we only studied 109 of the 300 tweets labeled by doctors. We excluded tweets where one of the doctors was unsure of the accuracy of the tweet or where there was disagreement between the two doctors regarding the accuracy of the tweet. These measures certainly excluded some health tweets and, more worryingly, may have thus excluded a class of health tweets that were not studied. However, the protocol did heighten the quality of the data in terms of its accuracy. In addition, all 20 doctors who participated in this study as annotators were from the same country, Saudi Arabia, the target of the study.

### Construction Validity

Although there was a high degree of agreement between the annotators who filtered out tweets not related to health, they did not have health backgrounds. This means that nonhealth tweets may have gotten through this phase. However, this was addressed when the doctors assessed the tweets for health accuracy; they did not identify any tweet as nonhealth related.

This study included the categorization of the authors of tweets into various groups; however, individual health accounts, health organization accounts, and individuals were not externally checked in order to test whether the classification was correct.

In addition, this study intended to examine tweets from Saudi users, specifically during the development of the health lexicon, which was noted in the study’s design. However, we cannot guarantee that all tweets had a Saudi origin.

### Conclusions

The purpose of this work was to validate the method used to ensure that it was practical for determining the accuracy and the factors associated with the trustworthiness and dissemination of health-related tweets; this was done to provisionally assess factors that may impact on trustworthy tweets and dissemination of tweets. Our results indicate that there may be some clear differences between tweets labeled as trustworthy health information and tweets labeled as untrustworthy health information. They also showed that trusted health professionals were more likely to tweet accurate health information, while exclusively SM-based accounts were more likely to produce untrustworthy tweets. Interestingly, most of the trustworthy tweets were tweeted in the morning, while more of the untrustworthy tweets were tweeted at night. Regarding the dissemination of tweets, there were some features that appeared to be associated with a high dissemination of the tweets. These features appeared at both the tweet-level and user-level analyses.

Due to the limited quantity of data, we cannot have confidence in statistical predictive modelling. The results illustrate that future studies using a large dataset may produce a predictive model for classifying tweets as either trustworthy or untrustworthy.

## Appendix

Multimedia Appendix 1 Complete list of keywords used in the study.

Multimedia Appendix 2 Tweet and user features used in the study.

## Abbreviations

API: application programming interface
CDC: US Centers for Disease Control and Prevention
F1: number of followers
F3: number of followees
FT1: number of tweets favorited by the author
FT2: number of times that author’s tweets are favorited
FT6: number of original tweets posted by the author that are favorited
LDH: local health department
M1: number of tweets where the author mentioned other users
M2: number of unique users mentioned by the author
MERS: Middle East respiratory syndrome
MH5: unique keyword count in hashtags set in original tweets posted by the author
MWW: Mann-Whitney-Wilcoxon
OT3: number of hashtags in the author‘s tweets
RM1: number of retweeted tweets by the author where the user mentioned other users
RMH5: unique hashtag count in tweets that were retweeted by the author
RMM5: unique mentions in retweeted tweets by the author
RP1: number of reply-to tweets posted by the author
RT1: number of tweets that the author retweeted
RT2: number of author’s tweets retweeted by other users
SM: social media
SSI: ratio of original tweets posted by the author to tweets retweeted by the author
TFF: ratio of followers to followees
