# Supplementary material for: Trustworthy Health-Related Tweets on Social Media in Saudi Arabia: Tweet Metadata Analysis
Source: J Med Internet Res. 2019 Oct 8;21(10):e14731. doi: 10.2196/14731 (PMC6914129; doi:10.2196/14731)
Supplement: Multimedia Appendix 1 [file jmir_v21i10e14731_app1.pdf]

إصابة

تصاب

مرض

شفاء

حماية

دواء

تحليل

عافية

ألم

إعياء

نقص

تسمم

أعراض

جرح

علاج

سلامة المرضى

مرض

العدوى

ملف المريض

الصحة

الأنظمة الصحية

السلامة الدوائية

علاج

فحص

أدوية عالية الخطر

التثقيف

استشارة الطبيب

تثقيف المرضى

قياس العلامات الحيوية

الأمراض النفسية

حقوق المرضى

أعراض

حساسي

الصرع

الكبد الوبائي

الإنزلاق الغضروفي

القولون

النقرس

قرحه الفم

الحمى الشوكية

الدسك

اكزيما

انسولين

التطعيم

الأرق

حساسيه

البواسير

الجلطة الوريدية

الصدفية

البهاق

فيتامين د

الرجفة

الأورام

صداع

القلق

حرقه المعدة

حمى المالطية

هشاشه العظام

الفصد

فيروس الكبد سي

تليف الكبد

علاج

مرض

صحه

انفلونزا

تشخيص

مرض السكري

كورونا

الكلى

المختبرات

التحاليل

فحص إكلينيكي

توعيه

سمنه

توحد

اكتئاب

ضغط الدم

سرطان

عسر الهضم

فقر الدم

السكتة الدماغية

الابهر

قسطرة

الربو

طفح جلدي

النزلة المعوية

الأدوية

هشاشه العظام

الغده الدرقية

عرق النسا

شرايين الجسم

القلب

السعال

كوليسترول

زهايمر

العمود الفقري

اكياس دهنية

حصى

الحامل

القرنية

زيكا

الطبيب

أعراض المرض

علاج المرض

مراجعة الطبيب

إسهال

استفراغ

تليف

زكمة

كحة

شعبي

التهاب

عدوى

بكتيريا

كورونا

السل

الزكام

قولون

الكبد

الكلى

صداع نصفي

مويا بيضاء

جلوكوما

نزيف

مخ

الدماغ

شوكية

ارتجاع

جيوب أنفية

قناة دمعية

طبلة الأذن

العصب

الدرقية

الغدة

الغدد

الليمفاوية

ليمفاوية

البنكرياس

الطحال

الرئة

القصبة الهوائية

البلعوم

لحمية

جراحية

بلازما

تشبع الأكسجين

نبض القلب

ضغط الدم

صمام قلب

قسطرة

الأورطى

الوريد

الأبهر

عملية

تدخل جراحي

كسر عظم

روماتيزم

هشاشة

نقص فيتامين

أيدز

ملاريا

الضنك

الحصبة

السعال

ألم مفاصل

احمرار

حكة

فشل كلوي

أنزيمات

الاثني عشر

دوالي

سرطان

علاج كيماوي

استئصال

الفقرات

غضروف

الحبل الشوكي

المريء

ربو

الربو

الحجاب الحاجز

حرقان

المعدة

فتق

المثانة

الحالب

حصوة كلى

تحليل دم

تحليل بول

جرثومة

سيلياك

حساسية القمح

نقرس

العيادة

المستشفى

أشعة صوتية

أشعة مغناطيسية

رنين مغناطيسي

أشعة مقطعية

أنيميا

منجلية

فقر دم

علاج طبيعي

المشيمة

الجنين

ارتفاع الضغط

ارتفاع السكر

قلبية

شلل نصفي

ارتعاشي
