# Supplementary material for: Trustworthy Health-Related Tweets on Social Media in Saudi Arabia: Tweet Metadata Analysis
Source: J Med Internet Res. 2019 Oct 8;21(10):e14731. doi: 10.2196/14731 (PMC6914129; doi:10.2196/14731)
Supplement: Multimedia Appendix 2 [file jmir_v21i10e14731_app2.pdf]

Multimedia Appendix 2

Tweet and user features used in the study

Level	Scope	Feature symbol	Description
Tweet	Activity	hashtags_count	Number of hashtags used in the tweet
Tweet	Activity	urls_count	Number of URLs used in the tweet
Tweet	Activity	URLs	Does the tweet contain URLs
Tweet	Activity	Hashtags	Does the tweet have hashtag or not
Tweet	Linguistic	count_words	Number of words in the tweet
Tweet	Linguistic	*Kashida*	Does the tweet contain *Kashida*
Tweet	Linguistic	charac_count	Number of characters in the tweet
Tweet	Linguistic	Exclamation_mark	Does the tweet contain '!'
Tweet	Linguistic	q_mark	Does the tweet contain '?'
Tweet	Linguistic	dot_statues	Does the tweet contain '.'
Tweet	Linguistic	Comma	Does the tweet contain '،'
Tweet	Linguistic	Semi_colon	Does the tweet contain '؛'
Tweet	Linguistic	Tashkeel	Does the tweet contain 'tashkeel'
Tweet	Popularity	Retweeted	Is the tweet retweeted or not
Tweet	Popularity	Fav	Is the tweet "favorited"
Tweet	Time	Time	Time at which the tweet is posted
Tweet	Time	week days	Is the tweet posted during week days or at the weekend

Level	Scope	Feature symbol	Description
User	Activity	wdeve_device	Most used device during the evening of the week days
User	Activity	wdmor_device	Most used device during the morning of the week days
User	Activity	wdnight_device	Most used type of device to post tweet during the night of the week days
User	Activity	wemor_device	Most used device during the morning of the weekend days
User	Activity	RT1	Number of tweets that author retweeted
User	Activity	OT1	Number of original tweets
User	Activity	Tweet Score	RT1 + OT1
User	Activity	RP1	Number of reply-to tweets posted by the author
User	Activity	FT1	Number of tweets this user has liked
User	Activity	GT	OT1+RP1
User	Activity	GT1	OT1+RP1+FT1
User	Activity	SSI	Rate of original tweets posted by the author to tweets retweeted by the author (OT1/RT1)
User	Activity	F3	Followees count

User	Activiety	OT2	Number of links shared
User	Activity	M1	Number of tweets where the author mentioned other users
User	Activity	M2	Number of unique users mentioned by the author
User	Activity	OT3	Number of hashtags in the author‘s tweets
User	Activity	MH6	Proportion of tweets that have hashtags to tweets that does not
User	Activity	GInSc	RT1+RP1
User	Activity	statuses_count	Number of tweets posted by the user
User	Activity	verified	Is the user account verified or not
User	Activity	location	Location of the user
User	Activity	mo_usedlang	Most used language of the user
User	Activity	se_mo_usedlang	Second most used language of the user
User	Activity	per_of_mo_usedlang	How often does the user use the most used language in their tweets?
User	Activity	per_of_se_mo_usedlang	How often does the user use the second most used language in their tweets?
User	Activity	n_countries	Number of countries visited by the author (if geo enabled)
User	Activity	n_cities	Number of cities visited by the author (if geo enabled)
User	Activity	MU1	Mean URL count in original tweets
User	Activity	MU2	Median URL count in original tweets
User	Activity	MU3	Max URL count in an original tweet
User	Activity	MU4	Minimum URL count in an original tweet
User	Activity	MU5	Unique URLs count in URLs in original tweets
User	Activity	RM1	Number of retweeted tweets by the author where the user mentioned other users
User	Activity	RMU1	Mean URL count in re-tweeted tweets
User	Activity	RMU2	Median URL count in re-tweeted tweets
User	Activity	RMU3	Max URL count in re-tweeted tweets
User	Activity	RMU4	Minimum URL count in re-tweeted tweets
User	Activity	MH1	Mean hashtags count in original tweets
User	Activity	MH2	Median hashtags count in original tweets
User	Activity	MH3	Max hashtags count in an original tweet
User	Activity	MH4	Minimum hashtags count in an original tweet
User	Activity	MH5	Unique hashtags count in original tweets
User	Activity	MM1	Mean mentions' count in original tweets
User	Activity	MM2	Median mentions count in original tweets
User	Activity	MM3	Max mentions count in an original tweet
User	Activity	MM4	Minimum mentions count in an original tweet
User	Activity	MM5	Unique mentions count in URLs in re-tweeted tweets
User	Activity	RMH1	Mean hashtags count in re-tweeted tweets
User	Activity	RMH2	Median hashtags count in re-tweeted tweets
User	Activity	RMH3	Max hashtags count in re-tweeted tweets

User	Activity	RMH4	Minimum hashtags count in re-tweeted tweets
User	Activity	RMH5	Unique hashtags count in tweets that were retweeted by the user
User	Activity	RMM1	Mean mentions' count in re-tweeted tweets
User	Activity	RMM2	Median mentions count in re-tweeted tweets
User	Activity	RMM3	Max mentions count in re-tweeted tweets
User	Activity	RMM4	Minimum mentions count in re-tweeted tweets
User	Activity	RMM5	Unique mentions in retweeted tweets by the author
User	Activity	rate_tweets_count_to_days	Average tweets per day
User	Activity	rate_GT_count_to_days	Average of general activity (post tweets, favourites) per day
User	**Activity**	trF1	Log transformed Friends
User	Activity	trF3	Log transformed Followers
User	Activity	trFT1	Log transformed Favourites
User	Activity	trstatuses_count	Log transformed Tweet count
User	Activity	OT1	Number of original tweets
User	Behaviour	geo_enabled	Is the geolocation enabled or not?
User	linguistic	UNC	Screen name words' count
User	linguistic	UDC	Description words' count
User	linguistics	lang	Language of the user
User	linguistics	n_langauges	Number of languages used by the user in their tweets
User	Popularity	F1	Followers count
User	Popularity	FR	F1/F1+F3
User	Popularity	TFF	F1/F3 (rate of followers to followees)
User	Popularity	RT5	Sum of retweet count for OT1 (tweets post by the user)
User	Popularity	RT2	Number of author’s tweets re-tweeted by other users
User	Popularity	FT2	Number of times author’s tweets are favorited
User	Popularity	FT6	Number of original tweets posted by the author that are "favorited"
User	Popularity	Listed_count	Number of lists where the user is member
User	Time	wd_freq	Rate of tweets posted during week days to total numbers of tweets
User	Time	wdeve_rate	Rate of tweets posted during evening of week days to total numbers of tweets
User	Time	wdmor_rate	Rate of tweets posted during morning of week days to total numbers of tweets
User	Time	wdnight_rate	Rate of tweets posted during night of week days to total numbers of tweets
User	Time	we_freq	Rate of tweets posted during weekend to total numbers of tweets
User	Time	weeve_rate	Rate of tweets posted during evening of weekend to total numbers of tweets
User	Time	wemor_rate	Rate of tweets posted during morning of weekend to total numbers of tweets
User	Time	wenight_rate	Rate of tweets posted during night of weekend to total numbers of tweets

User	Time	wenight_device	Most used type of device to post tweet during the night of the weekend
User	Time	twee_eve_we	Number of tweets posted by the user during evening of weekend
User	Time	twee_mor_we	Number of tweets posted by the user during morning of weekend
User	Time	twee_ni_we	Number of tweets posted by the user during night of weekend
User	Time	created_date	Date in which the user created the account
